# Three-dimensional printing and cochlear implantation: a systematic review

**DOI:** 10.1007/s00405-026-10563-8

**Published:** 2026-09-07

**Authors:** Eleonora M. C. Trecca, Annamaria Ruzza, Marco Cianca, Francesco di Pierro, Lazzaro Cassano, Giannicola Iannella, Alfonso Scarpa, Umberto Riccelli, Ciro Lucio Vigliaroli, Michele Cassano

**Affiliations:** 1 Department of Clinical and Experimental Medicine, Unit of Otorhinolaryngology- Head and Neck Surgery, University of Foggia, Viale Pinto, 1, 71122 Foggia, Italy; 2 Department of Otorhinolaryngology and Maxillofacial Surgery, IRCCS Hospital Casa Sollievo Della Sofferenza, San Giovanni Rotondo (Foggia), Italy; 3 Department of Otolaryngology-Head Neck Surgery, La Sapienza University, Rome, Italy; 4 Department of Medicine and Surgery, University of Salerno, Salerno, Italy

**Keywords:** Cochlear implants, Three-dimensional printing, Surgical training, Prosthesis design, Biocompatible materials, Otolaryngology

## Abstract

**Purpose:**

This systematic review aims to evaluate the applications of three-dimensional (3D) printing in cochlear implantation (CI), focusing on its roles in surgical training, pre-operative planning, intraoperative assistance, and device innovation.

**Methods:**

A systematic search of PubMed, Scopus, Web of Science, and Google Scholar was conducted from inception to August 30, 2025. Keywords included *“Cochlear Implant” OR “Cochlear Implantation” AND “3D printing”.* Additional articles were identified through manual cross-referencing. Eligible studies were original research articles in English that investigated the use of 3D printing in CI. Exclusion criteria included reviews, conference papers, and studies unrelated to CI. Reporting completeness was evaluated using the STROBE checklist, while methodological risk of bias was independently assessed using validated, domain-specific tools (ROBINS-I, SYRCLE, and adapted QUADAS-2 domains). Due to profound study heterogeneity, a narrative synthesis was performed.

**Results:**

Of the 76 publications identified, 22 studies met the inclusion criteria, with a marked increase after 2021. The included literature was divided into three functional areas: teaching models/surgical training (*n* = 13), preclinical research/device innovation (*n* = 3) and clinical/anatomical applications in humans (*n* = 6). The reported empirical accuracies ranged from 16 to 400 microns. The mean STROBE score was 14.8 ± 3.1, indicating moderate to high reporting quality. However, a rigorous methodological assessment revealed a high risk of bias among human and cadaveric series due to non-comparative designs and a lack of blinding on the part of the researchers, whilst studies using synthetic simulators demonstrated a predominantly low risk of bias.

**Conclusions:**

This review underscores the transformative potential of 3D printing in CI, particularly in enhancing surgical training and improving procedural outcomes. As the first systematic review focusing exclusively on cochlear-implant–specific applications, it addresses a critical gap in the literature and highlights opportunities for future research to standardize methodologies and explore long-term clinical impacts.

## Introduction

Recent advancements in cochlear implant (CI) technology have been driven by the integration of innovative tools such as three-dimensional (3D) printing, artificial intelligence (AI), and machine learning [1, 2]. These advancements have unlocked new possibilities for patient-specific modeling, surgical precision, and implant customization, enabling clinicians to provide tailored solutions to individuals with hearing loss. By converging engineering and medicine, 3D printing has emerged as a transformative tool in otologic surgery, facilitating improved clinical outcomes and advancing the boundaries of medical innovation [3].

For instance, Lei [4] introduced a co-modelling framework that utilizes 3D-printed biomimetic cochleae to interpret electric field imaging profiles of CI patients. This approach enhances understanding of patient-specific factors, paving the way for more effective and personalized implant strategies. Similarly, early research by Murugasu [5] demonstrated the potential of 3D rendering of magnetic resonance imaging (MRI) to create detailed models of the inner ear, which proved instrumental in guiding surgical decisions for CI. These foundational studies highlight the broader role of 3D imaging in otology, offering a glimpse into its transformative impact.

Over the years, the use of 3D printing in CI has transitioned from experimental to indispensable, encompassing applications in pre-operative planning, surgical training, intraoperative assistance, and device prototyping. Modern 3D printing technologies, including stereolithography, laser sintering, and multi-material extrusion, have enabled the creation of intricate anatomical models and custom surgical guides with unparalleled precision. The evolution of biocompatible materials, such as hybrid resins and silicone-like polymers, has further enhanced the realism and utility of 3D-printed models, making them integral to both educational and clinical settings [6].

One of the most significant contributions of 3D printing in CI is its role in surgical training. Anatomically accurate models of temporal bones and cochleae provide surgeons with hands-on opportunities to refine their techniques in a controlled, risk-free environment. These training models replicate the tactile and visual characteristics of human anatomy, reducing the learning curve for complex procedures and improving surgical proficiency [7].

In pre-operative planning, patient-specific 3D models derived from high-resolution imaging data [e.g., computed tomography (CT) and MRI scans] allow surgeons to visualize unique anatomical features and simulate complex procedures. This approach not only enhances surgical precision but also minimizes complications by offering a comprehensive understanding of the patient’s anatomy before surgery. Intraoperative tools, such as 3D-printed drill guides and electrode insertion templates, further improve accuracy during critical surgical steps [8].

Recent trends in CI have also emphasized the integration of 3D printing with other cutting-edge technologies, such as robotics [9]. These hybrid approaches aim to improve implant design, optimize surgical workflows, and enhance patient outcomes. For example, robotic-driven modeling combined with 3D printing could facilitate the development of adaptive CI devices capable of real-time feedback and fine-tuned electrode placement.

Moreover, 3D printing is playing a pivotal role in device prototyping and innovation. Rapid prototyping allows researchers to design and test electrode arrays and implantable components that align more closely with individual anatomical variations [10]. Biomimetic cochleae and 3D-printed molds have been used to assess insertion techniques and electrode design, accelerating the development of next-generation CI devices [11].

Looking ahead, advancements in biofabrication and tissue engineering hold promise for the use of 3D printing in regenerative medicine [12]. The development of bioengineered cochlear tissues and scaffolds could open new frontiers in the restoration of hearing, potentially offering alternatives to traditional CI devices.

This systematic review aims to provide a comprehensive analysis of the current applications of 3D printing in CI, including its role in surgical training, pre-operative planning, intraoperative procedures, and device innovation. By highlighting these advancements, this review underscores the transformative potential of 3D printing in improving outcomes for CI patients and discusses future directions in this rapidly evolving field.

## Materials and methods

### Study design and protocol

This systematic review was conducted in accordance with the Preferred Reporting Items for Systematic Reviews and Meta-Analyses (PRISMA) [13] guidelines. The review aimed to comprehensively evaluate the applications of 3D printing in CI, with a focus on its roles in surgical training, pre-operative planning, and intraoperative procedures. The review protocol was registered on the Open Science Framework (OSF) and is publicly available at https://doi.org/10.17605/OSF.IO/RD798**.**

### Eligibility criteria

Eligible studies were limited to English-language original research articles investigating the use of 3D printing specifically in CI procedure. Literature reviews, editorials, conference papers, and articles addressing 3D printing applications in other otologic surgeries were excluded. Studies involving cadaver, human, or animal models and providing sufficient methodological details were included.

### Search strategy

A systematic search was conducted in PubMed, Scopus, Web of Science, and Google Scholar databases from inception to August 30, 2025. The search terms used were: *(“Cochlear Implant” OR “Cochlear Implantation”) AND (“3D printing” OR “three-dimensional printing” OR “additive manufacturing” OR “rapid prototyping”)*. The search strategy was complemented by manual cross-referencing of relevant articles. Duplicate records were removed.

### Study selection

Two independent reviewers (AR and FDP) screened titles and abstracts to identify potentially relevant studies. Full texts of the shortlisted articles were then assessed against the inclusion and exclusion criteria. Disagreements were resolved through discussion by consulting a third reviewer (EMCT).

### Data extraction

Data were extracted independently by two reviewers (AR and FDP) using a pre-piloted data extraction form. Data regarding study characteristics (author, year, country, study design, sample size), 3D printing applications in CI procedure (e.g., surgical training, pre-operative planning, intraoperative uses), outcomes (e.g., accuracy, feasibility, educational impact) and methodology (e.g., materials used for 3D printing, imaging modalities, and printing technology) were collected.

For the extracted data, the concept of “accuracy” was operationally defined as the dimensional fidelity of the printed model relative to its digital design or reference anatomy, reported as a measured dimensional error (e.g., deviation in mm or µm assessed via caliper, microscopy, or micro-CT comparison). This was strictly distinguished from the printer’s nominal specifications (e.g., nominal XY resolution or layer thickness), which represent the machine’s capabilities rather than validated anatomical fidelity. Where the original studies reported only the printer’s nominal parameters without independent validation, these data were explicitly marked as not comparable to the empirical dimensional error.

### Quality assessment and risk of bias

To ensure a rigorous assessment of the included literature, a clear distinction was made between the quality of reporting and the risk of methodological bias. The completeness of reporting was assessed using the STROBE (Strengthening the Reporting of Observational Studies in Epidemiology) checklist. STROBE scores (ranging from 0 to 22) were used exclusively as indicators of reporting quality rather than methodological bias [14, 15].

The risk of methodological bias was assessed independently using validated, domain-specific tools adapted to the study designs identified in the review:

- Clinical and human cadaver studies: assessed using the Risk of Bias in Non-randomized Studies of Interventions (ROBINS-I) tool [16]. Seven domains were evaluated: bias due to confounding factors, participant selection, intervention classification, deviations from planned interventions, missing data, outcome measurement, and selection of reported outcomes.
- Animal studies: assessed using the Systematic Review Centre for Laboratory Animal Experimentation (SYRCLE) Risk of Bias tool [17], which covers sequence generation, baseline characteristics, allocation concealment, randomization, blinding (researchers and outcome assessors), outcome assessment, incomplete outcome data, and selective reporting.
- Synthetic phantoms and laboratory studies: since there is no universally accepted tool for purely engineering or educational studies on simulators, a transparent, domain-based checklist was adapted in accordance with the QUADAS-2 principles [18].

Each study was independently scored by two reviewers (EMCT and MC), and discrepancies were resolved by consensus.

### Data synthesis

A narrative review was conducted to summarize the main findings regarding the applications of 3D printing in cochlear implantation. Due to the marked heterogeneity of the study designs (ranging from synthetic bench-top models to preclinical animal studies and clinical case series), the varying clinical and technical outcomes (e.g., anatomical accuracy, insertion forces, educational acquisition, and imaging fidelity), and the inconsistent reporting of key quantitative parameters, a formal meta-analysis or an aggregated quantitative synthesis was deemed inappropriate and was not undertaken.

To systematically address this heterogeneity of evidence and improve conceptual clarity, the narrative review was structured hierarchically into three predefined functional areas:

1. Educational models and surgical training: including synthetic bench-top manikins and laboratory simulators.
2. Preclinical research and device innovation: including animal studies and engineering prototyping.
3. Clinical and human anatomical applications: including patient-specific surgical planning and validation of intraoperative guides on human cadavers.

Quantitative data extracted from the included articles were limited exclusively to descriptive statistics, including absolute counts and proportions to summarize study characteristics (e.g., publication trends over time, distribution of 3D printer types, and categories of manufacturing materials). Where physical parameters such as print accuracy or dimensional error were reported, these were summarized using ranges (minimum and maximum values) to present the current state of the technology without making direct statistical comparisons.

## Results

Given the diverse nature of the studies identified, no statistical synthesis of the evaluation parameters was performed. Instead, a narrative approach, supplemented by descriptive indicators, was adopted to map the applications of 3D printing in the field of CI.

**Fig. 1:**
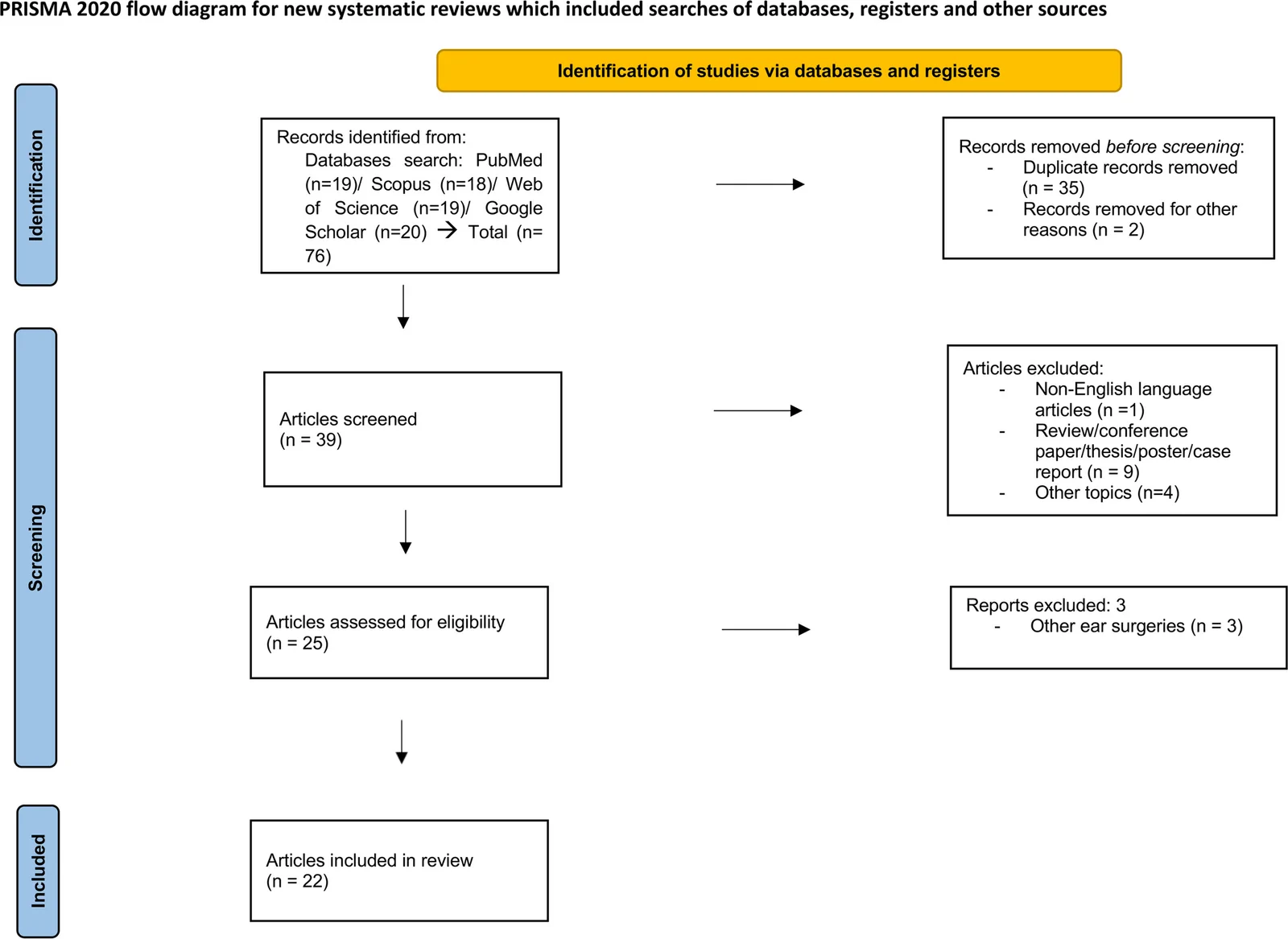
Preferred Reporting Items for Systematic Reviews and Meta-Analyses (PRISMA) flow chart

The systematic search identified 76 records from PubMed, Scopus, Web of Science, and Google Scholar. After removing duplicates (*n* = 35) and two additional records (*n* = 2), 39 articles underwent screening. Of these, 14 records were excluded for not meeting the eligibility criteria, including non-English language articles (*n* = 1), review/conference papers, or theses, posters (*n* = 9) and manuscripts unrelated to 3D printing and cochlear implantation (*n* = 4). Following a detailed eligibility assessment of 25 articles, 3 studies focusing on other ear surgeries were excluded, leaving 22 articles [4, 10, 19–39] for inclusion in the final analysis (Fig. 1; Table 1).

**Table 1:** Summary of included studies on 3D printing in cochlear implantation

FIRST AUTHOR	TITLE	YEAR	COUNTRY	Study population	Type of study	Number of patients/animal/specimens	Animal model	Cadaver study (n)	Use of 3D printing/CI	3D printing type	3D printing material	Accuracy	STROBE SCORE*
*Aregueta Robles U*	Growing human-scale scala tympani-like in vitro cell constructs	2023	Australia	Synthetic	Prospective	3	N/A	Scala tympani	Training	Stereolitography	Polymeric (PVA-PBS)	> 100 µm	14
*Banu C*	Virtual 3-Dimentional Models of Abnormal Cochleae for Cochlear Implantation	2017	Malaysia	Synthetic	Prospective	2	N/A	N/A	Training	Stereolitography (Stratasys)	Polymeric (Vero: = rigid material for bone; Tango: flexible material with rubber-like qualities)	N/A	14
*Bautista Salinas D*	Integrated Augmented Reality Feedback for Cochlear Implant Surgery Instruments	2020	United Kingdom	Synthetic	Retrospective	2	N/A	Temporal bone	Intraoperative	Stereolitography	Polymeric (Vero = rigid material for bone; ABS-M30i: bio-compatible plastic material)	127 µm	11
*Böttcher-Rebmann G*	Universal test bench for repeatable multiparametric cochlear implant insertion tests	2023	Germany	Synthetic	Prospective	3	Porcine	Temporal bone (cochleae)	Training: testing novel electrode array designs, insertion tools, or techniques	Stereolitography	N/A	N/A	8
*Canzi P*	Customized Cochlear Implant Positioning in a Patient With a Low- Grade Glioma: Towards the Best MRI Artifact Management	2022	Italy	Human	Prospective	1	N/A	Temporal bone (Patient-specific 3D printed head model)	Preoperative/improved outcomes	Stereolitography (Objet 260 Connex 3, stratasys)	Photopolymer	N/A	13
*Cramer J*	Temporal bone phantom for decoupled cochlear implant electrode insertion force measurement	2023	Germany	Synthetic	Prospective	1	N/A	Temporal bone + cochlea	Training: testing novel electrode array designs, insertion tools, or techniques	PolyJet 3D printer (Objet 350 Connex3, Stratasys, USA)	Polymers (Vero = rigid material for bone; Agilus30 = silicone-like substance for skin and soft tissues)	N/A	14
*Cramer J*	A method for accurate and reproducible specimen alignment for insertion tests of cochlear implant electrode arrays	2024	Germany	Animal	Prospective	15	Porcine	Animalhead	Training	Laser	PSA 3D printer	100 µm	18
*Gautam I*	Creation of a Prototype Cochlear Training Model	2024	United States	Synthetic	Prospective	1	N/A	Temporal bone (cochlea)	Training/improved outcomes	N/A	VeroClear (a transparent PolyJet photopolymer)	N/A	12
*Hrncirik F*	Impact of Scala Tympani Geometry on Insertion Forces during Implantation	2022	United Kingdom	Human	Retrospective	1	N/A	Scala tympani	Training/improved outcomes	Stereolitography	Clear Microfluidics Resin	30 µm	16
*Isaakidou A*	Multi-scale in silico and ex silico mechanics of 3D printed cochlear implants for local drug delivery	2024	Netherlands, Switzerland, United States	Synthetic	Retrospective	3	N/A	Scala tympany	Training	Stereolitography	Resin (IP-Q™: nanoscribe, Eggenstein-Leopoldshafen, Germany)	50 µm	11
*Jiang C*	An Instrumented Cochlea Model for the Evaluation of Cochlear Implant Electrical Stimulus Spread	2021	United Kingdom, Canada	Synthetic	Prospective	1	N/A	Temporal bone	Training	Stereolitography (Formlabs Form 2)	Polymeric (electrically-insulating methacrylate resin and ultraviolet (UV)-cured)	N/A	18
*Lei I. M*	3D printed biomimetic cochleae and machine learning co-modelling provides clinical informatics for cochlear implant patients	2021	Multicentric	Human	Prospective	82	N/A	Biomimetic cochleae	Improved outcomes	Stereolitography	Multimaterial robotic bioprinter	400 µm	15
*Leon L*	Scala-Tympani Phantom With Cochleostomy and Round-Window Openings for Cochlear-Implant Insertion Experiments	2014	USA	Synthetic	Prospective	2	N/A	Temporal bone	Training testing novel electrode array designs, insertion tools, or techniques	(Micro)Stereolitography	Plastic (watershed)	140–280 µm	16
*Markodimitraki L. M*	Cochlear implant positioning and fixation using 3D-printed patient specific surgical guides; a cadaveric study	2022	Netherlands	Human	Prospective	9	N/A	Temporal bone	Training	Laser	Photopolymer resin	300 µm	13
*Micault E*	Preclinical Study of a Minimally Invasive Cochlear Implant Device Based on 3D Printing of a Custom Guide	2025	France	Human	Prospective	8	N/A	Temporal bone	Custom minimally invasive surgical guide for CI insertion	Stereolithography	Biocompatible photopolymer resin	200 µm	20
*Morrel W. G*	Custom mastoid-fitting templates to improve cochlear implant electrode insertion trajectory	2020	USA	Human	Prospective	7	N/A	Temporal bone	Intraoperative/improved outcomes	Stereolitography	Biocompatible resin (Dental SG resin, Formlabs Inc)	N/A	14
*Rau T. S*	A method for image-guided positioning of cochlear specimens in insertion test benches using 3D printed stands	2021	Germany	Animal	Prospective	1.0	Porcine	Temporal bone (cochleae)	Training: testing novel electrode array designs, insertion tools, or techniques	Stereolitography (3D printer—Ultimaker 2, Netherlands)	Resin	23 µm	14
*Rienas W*	3D printed temporal bones for preoperative simulation and planning	2024	United States	Human	Prospective	5	N/A	Temporal bone (CT scan)	Simulation training, surgical planning and use intraoperatively in temporal bone surgery	Stereolitography (Projet 5500 × -E 3D printer)	CE-NT amber soft resin for the facial nerve and cho- lesteatoma, and CR-CL hard translucent resin for bony structures	32 μm	20
*Smetak M. R*	Beyond the phantom: unroofing the scala vestibuli in a fresh temporal bone as a model for cochlear implant insertion experiments	2022	USA	Synthetic	Prospective	5	N/A	Temporal bone	Training: testing novel electrode array designs, insertion tools, or techniques	Stereolitography (3D printer—Formlabs, Somerville, Massachusetts)	Resin (epoxy)	N/A	15
*Starovoyt A*	Anatomically and mechanically accurate scala tympani model for electrode insertion studies	2023	Belgium	Human	Prospective	2	N/A	Temporal bone/human cadaveric cochleae	Training	Stereolitography (Objet Connex 350 3D Printer: Stratasys, Eden Prairie, MN, USA)	Polymers (Vero = rigid material for bone)	16 µm	19
*Swaddiwudhipong N*	Investigating the Electrical Properties of Different Cochlear Implants	2021	UK, Canada	Synthetic	Prospective	4	N/A	Biomimetic cochleae	Training: testing novel electrode array designs, insertion tools, or techniques	Stereolitography (3D printer—Formlabs, Somerville, Massachusetts)	Resin (methacrylate)	250 µm	17
*Wei C*	Development of a drug delivering round window niche implant for cochlear pharmacotherapy	2024	Germany, China	Animal	Retrospective	37	Porcine	Temporal bone/round window	Training	Stereolitography (3D-Bioplotter® Manufacturers series)	Silicone	200 µm – 400 µm	13

Abbreviations: *3D* indicates three-dimensional, *STROBE* Strengthening the reporting of observational studies in epidemiology, *µm* micron, *N/A* not applicable, *O.C.T* optimal cutting temperature, *PVA – PBS* polyvinyl alcohol—phosphate buffer solution, *PSA* pose setting adapters, *PDMS* Polydimethylsiloxane, *Scores interval from 0 to 22, with higher scores showing better study quality

**Fig. 2:**
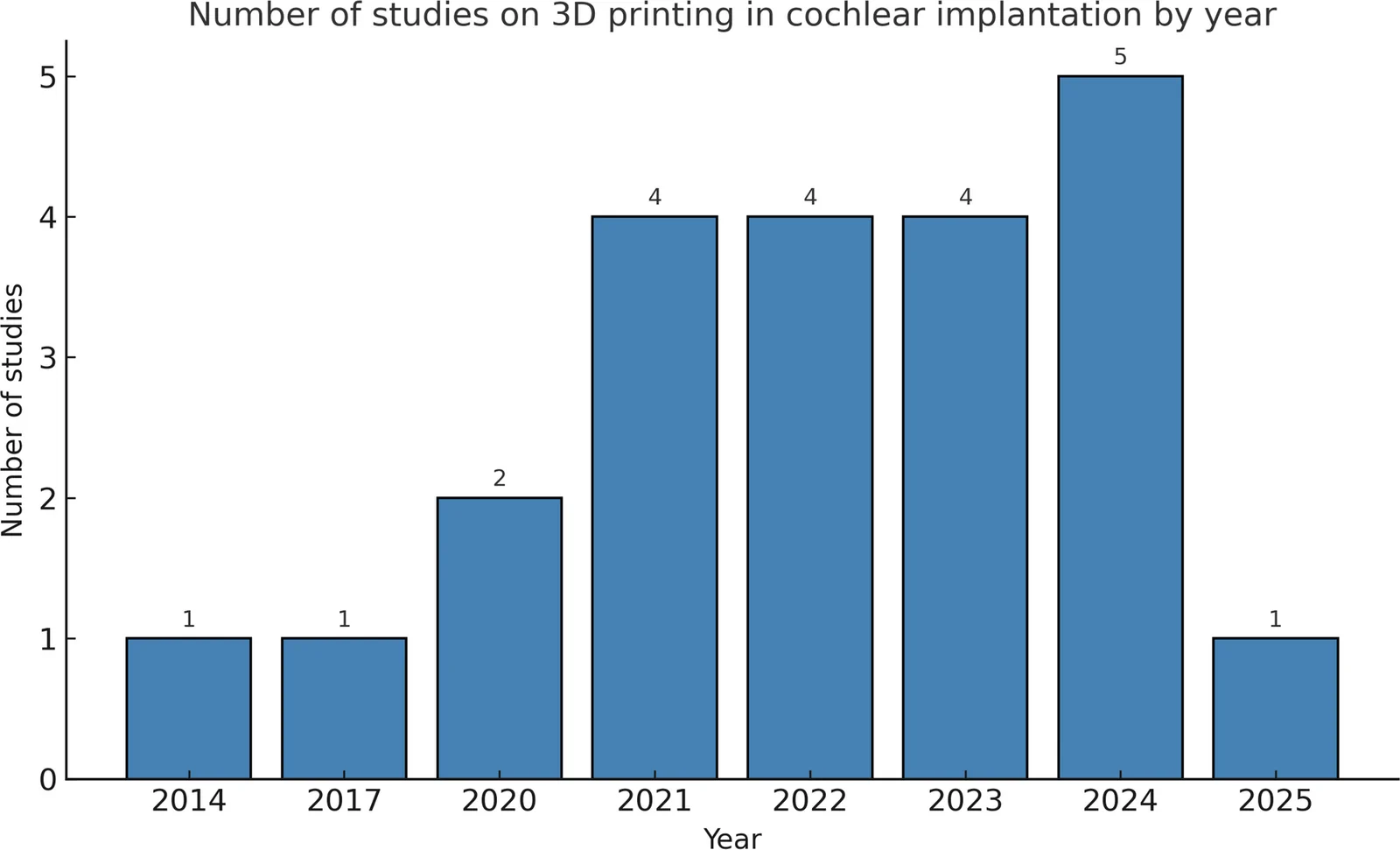
Number of studies on 3D printing in cochlear implantation by year

**Fig. 3:**
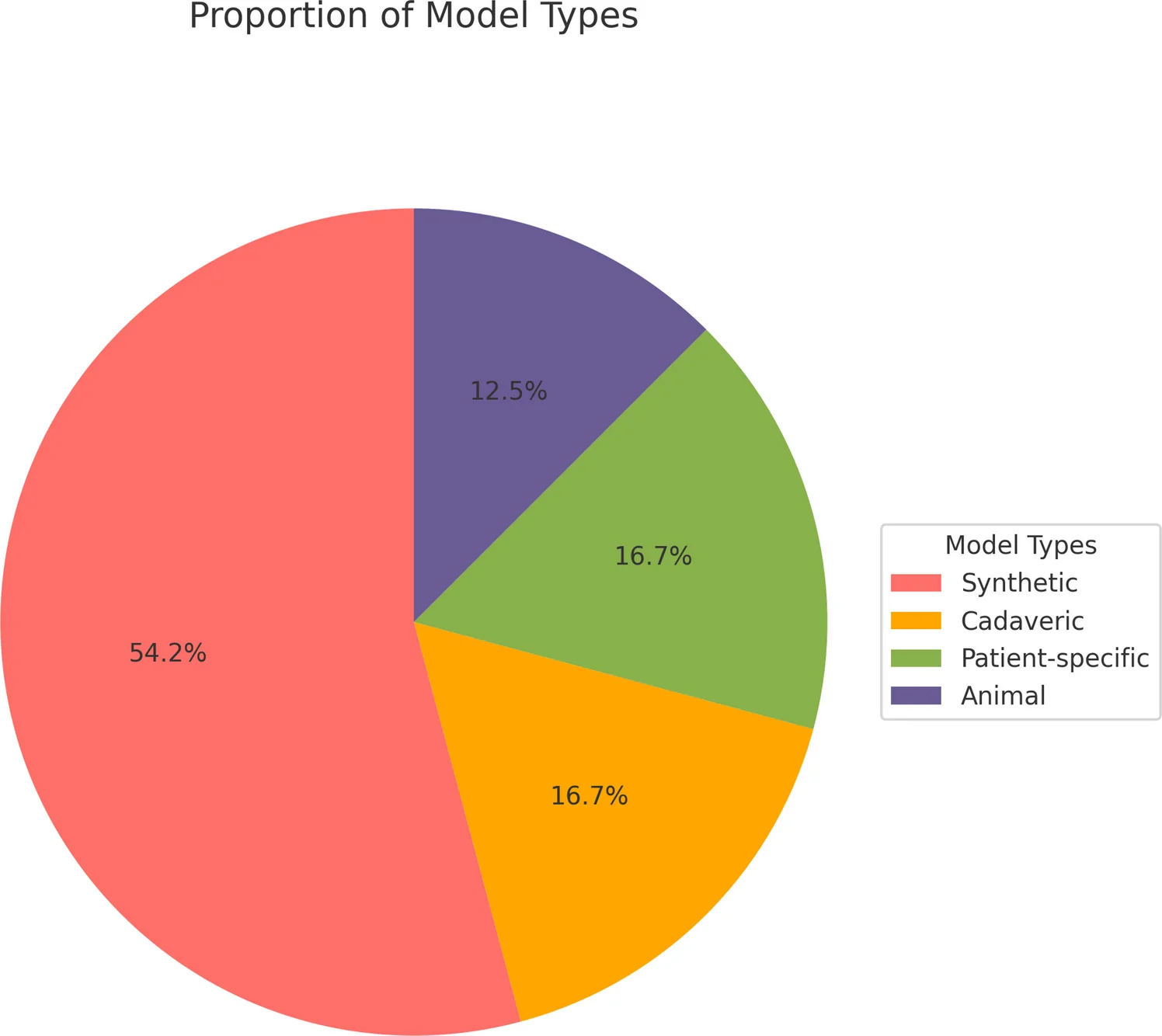
Pie chart showing the proportions of study models (e.g., cadaveric, animal, synthetic, human)

**Fig. 4:**
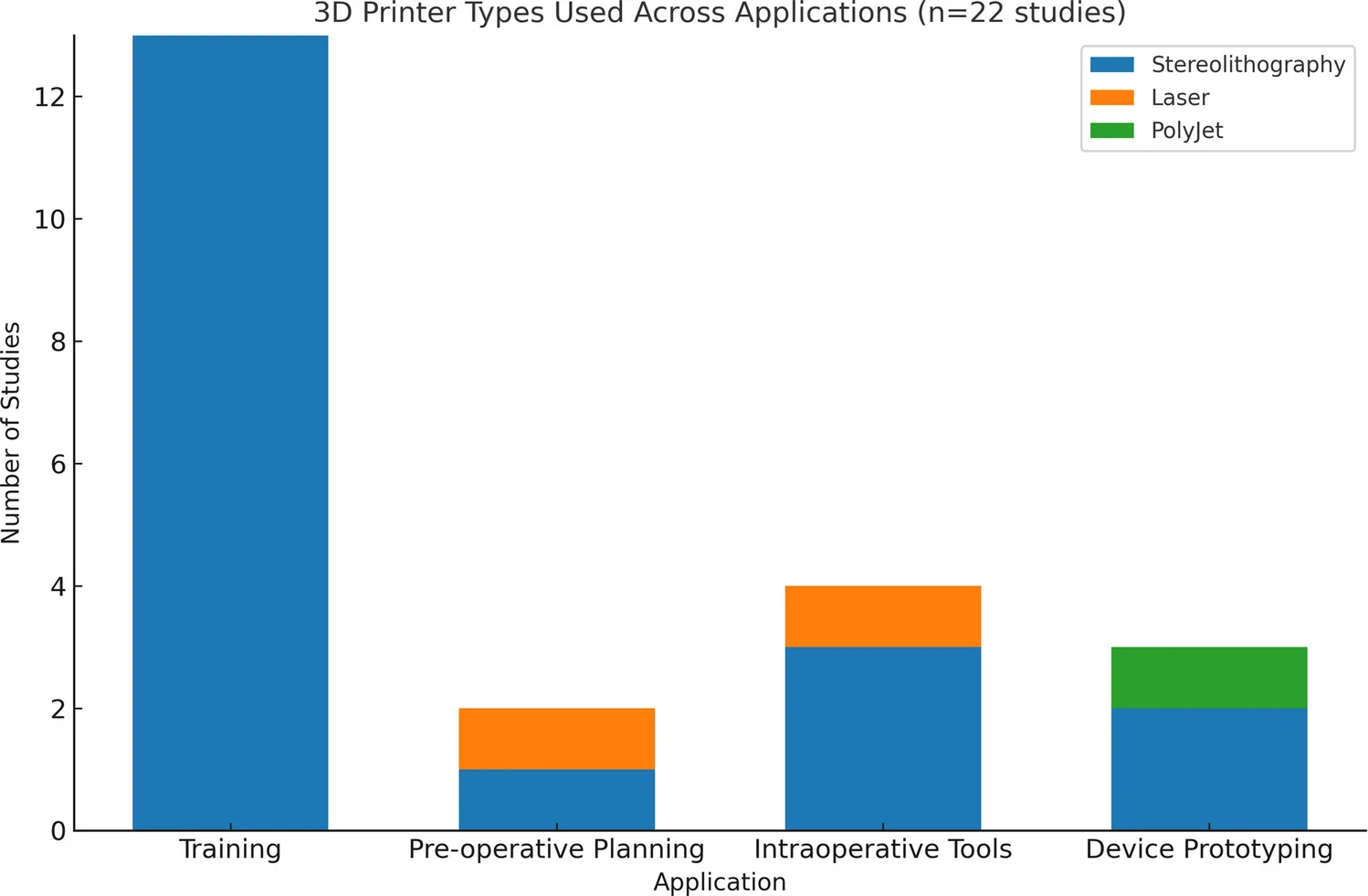
Stacked bar chart showing 3D printer types used across applications

These studies were published between 2017 and 2025**,** with a marked increase in publications over the last four years; more than 80% were published after 2021, evidencing the growing research interest in this field (Fig. 2). Most studies originated from Europe and North America. This trend reflects increasing advancements in 3D printing technologies, aligning with its growing adoption in medical fields (Figs. 3 and 4).

**Fig. 5:**
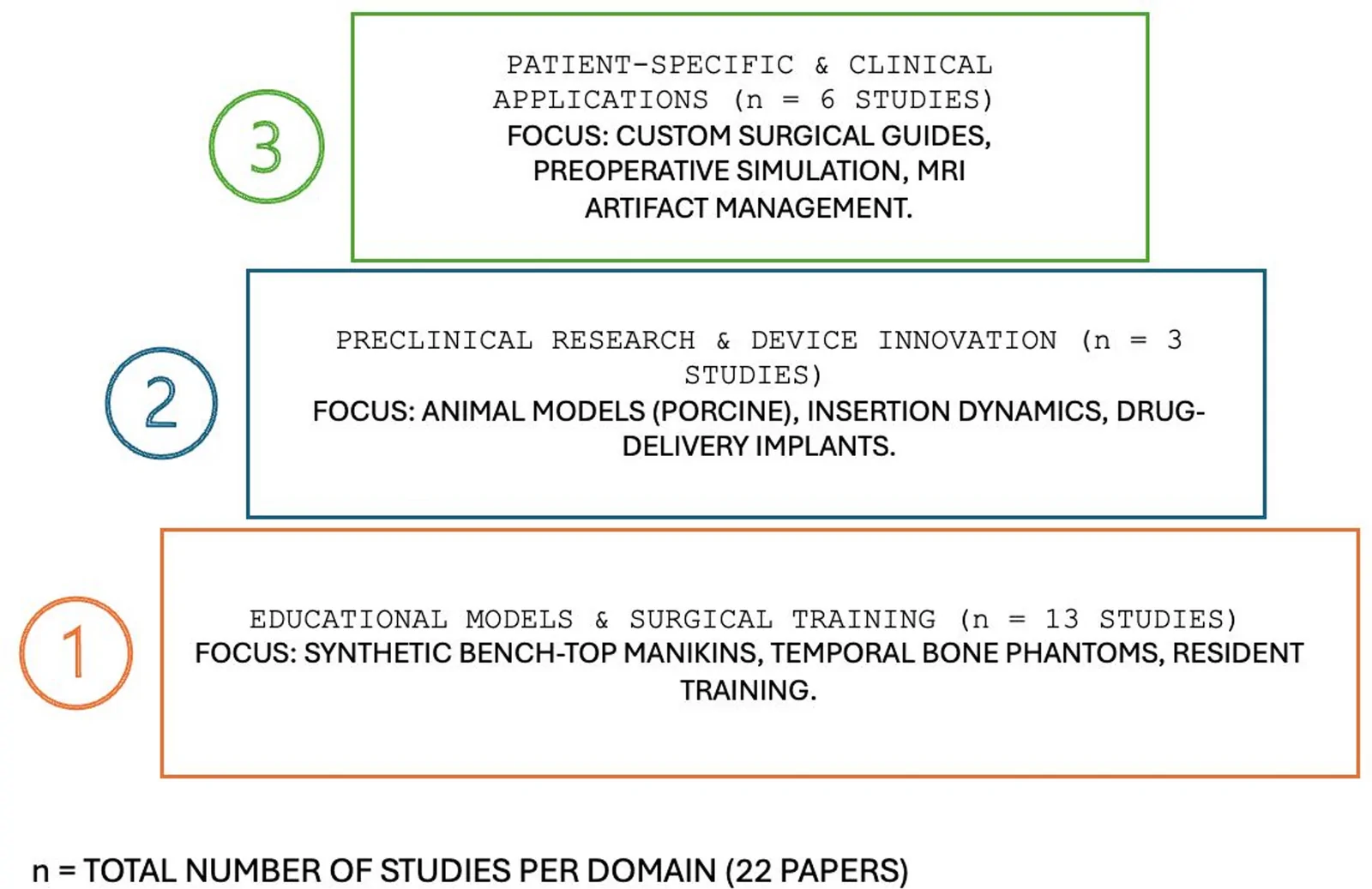
Evidence hierarchy of included studies according to translational maturity, ranging from educational simulation models to patient-specific clinical applications

The included articles highlighted various applications of 3D printing in cochlear implantation, which were divided into three distinct methodological and functional categories to account for the high heterogeneity of the evidence base: surgical training, preclinical research and device innovation, as well as clinical applications (Fig. 5).

In these areas, the studies have used various types of models, including synthetic manikins [4, 10, 21–23, 25–27, 29, 32, 33, 35, 36] (*n* = 13), animal models [24, 28, 29] (*n* = 3), and human specimens [19, 30, 31, 31, 37, 40] (*n* = 6) (Fig. 3).

### Educational models and surgical training

Overall, most of the literature applied 3D printing primarily for educational and surgical simulation purposes [10, 21–26, 32–35, 39, 41] (*n* = 13; 59.1%). This area relied predominantly on synthetic bench-top manikins and laboratory simulators (such as those by Aregueta Robles et al. [35], Banu et al. [32], and Smetak et al. [22]). All 13 training studies employed stereolithography-based 3D printing, highlighting its dominance as the go-to technology for producing anatomical manikins and temporal bone models (Fig. 4). The accuracies reported in this subgroup were moderately precise (e.g., up to 210 microns), optimizing the reproduction of the macro-anatomy of the temporal bone and the spaces of the middle ear necessary for the practical training of residents.

### Preclinical research and device innovation

This intermediate category included studies focused on prototyping, evaluating insertion dynamics, and implementing advanced drug delivery systems [4, 27, 28] (*n* = 3; 13.6%). Animal models, primarily pigs (*n* = 3), provided anatomically and biologically relevant conditions for testing new surgical approaches, optimizing electrode array design, and validating niche implants for microscale drug delivery. The largest experimental series in this preclinical subgroup was that of Wei [28] et al., who tested 37 porcine models to evaluate round-window niche pharmacotherapy.

### Clinical applications and human anatomy

Studies involving real-world clinical applications and those on high-fidelity human anatomy accounted for 6 articles in the narrative review. However, considering the basic characteristics of the materials used, studies involving human biological samples across the entire review accounted for 8 articles, equally divided between cadaver models [19, 34, 37, 40] (*n* = 4) and patient-specific models [4, 30, 31, 33] (*n* = 4) (Fig. 3).

These studies focused on intraoperative assistance [19, 36, 37, 40] (*n* = 4; 18.2%) and patient-specific preoperative planning [30, 31] (*n* = 2; 9.1%). Key applications included custom, patient-specific surgical guides; preoperative simulation of complex temporal bone anomalies using models derived from high-resolution CT scans; and artifact management in postoperative imaging. Sample sizes in this category were generally small, reflecting proof-of-concept designs (≤ 5 samples), with the notable exception of Lei [4] et al., who included 82 cases of biomimetic cochleae. Manufacturing in this clinical subgroup required the highest precision, with reported dimensional tolerances reaching 16–30 microns to accurately capture complex inner ear structures such as the scala tympani.

Common materials included resin, multimaterial systems, and PSA-compatible materials, with stereolithography and laser-based printing technologies being predominant. All training studies (*n* = 13) employed stereolithography-based 3D printing, underscoring its dominance as the reference technology for producing anatomical phantoms and temporal bone models (Fig. 4). Reported accuracies ranged from 16 to 400 microns, with higher precision models (e.g., 25 microns) used for intricate anatomical structures like the cochlea and moderate precision (e.g., 210 microns) applied to training models.

The quality of reporting, as measured by the STROBE score, had a mean value of 14.8 ± 3.1 (range: 8/22 to 20/22), indicating an overall standard of reporting completeness ranging from moderate to high across the included literature.

However, rigorous assessment of the risk of methodological bias revealed significant difficulties across various domains of evidence (Table 2).

**Table 2:** Methodological risk of bias assessment

Study (Author, Year)	Confounding	Selection	Intervention Class		Deviations	Missing Data	Outcome Measurement	Reported Result		Overall RoB
Canzi (2022)	High	Moderate	Low		Low	Low	Moderate	Moderate		**Serious**
Hrncirik (2022)	High	Moderate	Low		Low	Low	Moderate	Moderate		**Serious**
Lei (2021)	Moderate	Moderate	Low		Low	Low	Moderate	Moderate		**Moderate**
Markodimitraki (2022)	High	Moderate	Low		Low	Low	Moderate	Moderate		**Serious**
Micault (2025)	High	Moderate	Low		Low	Low	Moderate	Moderate		**Serious**
Morrel (2020)	High	Moderate	Low		Low	Low	Moderate	Moderate		**Serious**
Rienas (2024)	High	Moderate	Low		Low	Low	Moderate	Moderate		**Serious**
Starovoyt (2023)	High	Low	Low		Low	Low	Moderate	Moderate		**Serious**
Study (Author, Year)	Sequence Gen	Baseline Char	Alloc. Conceal	Random Housing	Blinding (Opr.)	Random Outcome	Blinding (Eval.)	Incomplete Data	Selective Report	Overall RoB
Cramer (2024)	Unclear	Low	Unclear	Unclear	High	Unclear	Moderate	Low	Moderate	**Unclear**
Rau (2021)	Unclear	Low	Unclear	Unclear	High	Unclear	Moderate	Low	Moderate	**Unclear**
Wei (2024)	Unclear	Low	Unclear	Unclear	High	Unclear	Moderate	Low	Moderate	**Unclear**
Study (Author, Year)	Model/Specimen Selection	Conduct/Performance Bias				Outcome Measurement		Selective Reporting		Overall RoB
Aregueta Robles (2023)	Some Concerns	Low Risk				Low Risk		Low Risk		**Low Risk**
Banu (2017)	Some Concerns	Low Risk				Low Risk		Low Risk		**Low Risk**
Bautista Salinas (2020)	Some Concerns	Low Risk				Low Risk		Low Risk		**Low Risk**
Böttcher-Rebmann (2023)	Low Risk	Low Risk				Low Risk		Low Risk		**Low Risk**
Cramer (2023)	Low Risk	Low Risk				Low Risk		Low Risk		**Low Risk**
Gautam (2024)	Some Concerns	Low Risk				Low Risk		Low Risk		**Low Risk**
Isaakidou (2024)	Low Risk	Low Risk				Low Risk		Low Risk		**Low Risk**
Jiang (2021)	Some Concerns	Low Risk				Low Risk		Low Risk		**Low Risk**
Leon (2014)	Low Risk	Low Risk				Low Risk		Low Risk		**Low Risk**
Smetak (2022)	Some Concerns	Low Risk				Low Risk		Low Risk		**Low Risk**
Starovoyt (2023)	Low Risk	Low Risk				Low Risk		Low Risk		**Low Risk**
Swaddiwudhipong (2021)	Low Risk	Low Risk				Low Risk		Low Risk		**Low Risk**

Among the series involving human subjects and cadavers (*n* = 8), the overall risk of bias was predominantly classified as serious. This assessment was primarily driven by a high risk of confounding due to the non-comparative and descriptive nature of the included case series and case reports, along with a moderate to high risk in outcome measurement resulting from the lack of researcher blinding during accuracy and trajectory assessments. Only the largest clinical cohort study (Lei et al. [4], *n* = 82) showed a moderate overall risk due to its large sample size and standardized data collection.

For animal models (*n* = 3), the risk of bias was classified as “Unclear.” Although the baseline characteristics of the pig models were highly standardized (low risk), details regarding sequence generation, allocation concealment, and randomization of subjects were systematically omitted or inadequately reported in the original texts. Furthermore, blinding of the surgical operators was inherently unfeasible due to the nature of the procedures.

In contrast, the educational synthetic bench-top and simulator studies (*n* = 13) showed a predominantly low risk of methodological bias. These investigations demonstrated high technical proficiency, utilizing calibrated force sensors, micro-CT validation, and standardized automated test benches, which minimized performance and detection biases. A minor concern (“Some concerns”) was noted regarding sample selection, as most digital models were derived from convenience samples of specific patient anatomies rather than randomized anatomical variations.

## Discussion

This systematic review analyzed 22 studies investigating the applications of 3D printing in CI, providing a comprehensive overview of its role in surgical training, pre-operative planning, intraoperative guidance, and device innovation. Overall, the evidence highlights the increasing adoption of additive manufacturing in otologic surgery, reflecting the broader trend of precision medicine and personalized surgical strategies in the last years. While there are existing reviews on the use of 3D printing in otolaryngology and otology more broadly [42], which have previously included cochlear implantation among multiple indications, the present study represents the first effort to systematically synthesize evidence focusing exclusively on CI-specific applications, establishing a dedicated study-domain hierarchy.

### Educational models and surgical training

Most included studies employed 3D-printed temporal bones or cochlear models as educational tools. Starovoyt et al. [34] demonstrated the anatomical and mechanical fidelity of resin-based scala tympani models, reporting micrometric precision that allowed reliable simulation of insertion mechanics. Similarly, Smetak et al. [22] and Cramer et al. [26, 29] validated the use of synthetic models for quantifying electrode insertion forces, offering reproducible platforms for training and research. Böttcher-Rebmann et al. described a universal test bench based on porcine temporal bones that enables repeatable, multiparametric insertion testing, thus bridging synthetic and animal-based approaches [25]. These models provide tactile feedback and visual realism that surpass 2D imaging or virtual simulations, thereby shortening the learning curve for junior surgeons and enabling experienced surgeons to refine techniques in rare or complex anatomies.

### Clinical and human anatomical applications

Several studies emphasized the clinical benefits of patient-specific 3D-printed models. Canzi et al. [31] reported the successful use of customized CI positioning to reduce MRI artifacts in a patient with a low-grade glioma. Morrel [19] and Markodimitraki et al. [37] demonstrated the feasibility of surgical guides tailored to individual mastoid anatomy, which improved electrode insertion trajectories and fixation stability. Rienas et al. [30] further highlighted the role of CT-derived temporal bone models for preoperative planning, simulation, and intraoperative reference. Although most of the research is still in the preclinical phase, these applications provide a valuable framework for feasibility testing that allows for the anticipation of anatomical challenges in patients with congenital malformations, cochlear ossification, or a history of previous otologic surgery. While authors often hypothesize that such models can minimize intraoperative uncertainty or shorten operating times, these potential clinical benefits remain speculative due to the lack of comparative clinical studies.

From a technical standpoint, 3D-printed drill guides, mastoid templates, and insertion tools were shown to enhance procedural accuracy. Morrel et al. [19] found that custom mastoid-fitting templates optimized insertion angles, potentially reducing trauma to cochlear structures. Similarly, Markodimitraki et al. [37] demonstrated cadaveric feasibility of patient-specific guides, which suggests that the research is feasible; however, the transition from experimental application to routine clinical practice remains heavily dependent on long-term clinical validation and overcoming significant hurdles relating to the biocompatibility of materials and workflow.

More recently, Micault et al. [40] conducted a preclinical cadaveric study evaluating a minimally invasive CI device guided by a custom 3D-printed template. Their results confirmed the feasibility and accuracy of this approach, supporting the potential clinical translation of patient-specific insertion guides.

### Preclinical research and device innovation

The review also revealed substantial progress in CI device research. Lei et al. [4] pioneered biomimetic cochleae combined with machine learning, enabling individualized predictions of electrical field distribution and facilitating electrode design optimization. Starovoyt et al. [34] and Hrncirik et al. [33] showed that 3D-printed scala tympani phantoms can model insertion forces, allowing comparative testing of electrode stiffness and geometry. More recently, Isaakidou et al. [27] explored multi-scale printed CI with drug-delivery potential, while Wei et al. [28] developed a niche implant for pharmacotherapy, highlighting how 3D printing may expand CI beyond auditory stimulation towards combined electro-acoustic and pharmacological rehabilitation.

The integration of 3D printing with emerging technologies such as robotics, augmented reality, and computational modeling [43–45] further expands its potential. Bautista-Salinas et al. [36] integrated augmented reality with 3D-printed surgical tools, underscoring the potential for hybrid approaches combining visualization technologies with physical models, while Shoman [9] emphasized the synergy between robotics and patient-specific planning in reducing human error. Such integration suggests a future in which 3D printing may not only support preoperative and intraoperative steps but also become part of adaptive, feedback-driven surgical ecosystems.

Although this review is focused on CI, parallels can be drawn from other otologic applications. For instance, Iannella et al. [7] validated the “SAPIENS” model for middle ear education, and Della Volpe et al. [8] used 3D reconstructions for a Bonebridge transcutaneous bone conduction implant (MED-EL, Innsbruck, Austria) planning. Similarly, progress has been made in the endoscopic domain of ear surgery, where other authors validated multimaterial 3D-printed simulators for training in tympanoplasty and endoscopic stapes surgery [46, 47]. These contributions highlight how 3D printing is extending its educational and clinical applications, further supporting its cross-disciplinary scalability in different fields of otology and auditory implants.

From a clinical standpoint, the implications of the findings of this systematic review are twofold. First, 3D printing is already a tangible resource for otologic surgeons in the fields of surgical training and pre-operative rehearsal. Anatomically faithful temporal bone and cochlear models provide a safe environment for residents and junior surgeons to practice electrode insertion, and for senior surgeons to prepare for rare or complex anatomies such as cochlear ossification or inner ear malformations [22, 29, 34]. Patient-specific planning models, although limited to a few reports, represent a feasible adjunct in challenging cases [30, 31]. Second, intraoperative applications (e.g., drill guides and insertion templates) and pharmacological innovations (e.g., drug-delivering round window implants) remain experimental, with technical barriers such as sterilization, production time, and cost still preventing routine use. [19, 27, 28, 37]. Additional translational barriers should also be acknowledged. Patient-specific surgical guides and implant-related adjuncts may require dedicated regulatory approval pathways depending on their intended use and jurisdiction. Material biocompatibility, sterilization compatibility, traceability, and quality-management requirements must be addressed before widespread clinical implementation. Furthermore, workflow integration remains challenging because segmentation, virtual planning, manufacturing turnaround times, and interoperability with navigation, robotic, or augmented-reality platforms may substantially increase procedural complexity. Finally, surgeon-dependent variability, institutional expertise, and cost-effectiveness considerations may limit scalability and contribute to the gap between promising experimental findings and routine clinical practice. To date, clinical studies conducted on human cadavers or specific patients have resulted in 8 articles (including the series by Canzi et al. [31], Hrncirik et al. [33], Lei et al. [4], Rienas et al. [30], Morrel et al. [19], Markodimitraki et al. [37], Micault et al. [40], and Starovoyt et al. [34]), highlighting that direct translation into routine clinical practice remains limited.

For ear surgeons, therefore, 3D printing should currently be regarded as an excellent tool for training and preoperative simulation. The actual benefits for patients and its clinical superiority over conventional approaches have yet to be demonstrated, and well-designed comparative studies will be needed to establish whether its theoretical advantages can translate into measurable improvements in clinical outcomes.

### Limitations of current evidence and future perspective

Despite promising results, the included studies presented heterogeneity in design, sample size, and outcome measures. Accuracy ranged widely (16–400 µm), and materials varied from rigid resins to silicone-like polymers, complicating comparisons. Most investigations were proof-of-concept, cadaveric, or animal-based, with limited clinical validation and absence of randomized controlled trials. Furthermore, long-term outcomes, such as implant survival, speech perception, and cost-effectiveness, remain largely unexplored. Sterilization protocols, regulatory pathways, and integration into hospital workflows also require standardization before routine clinical adoption.

Cost–benefit considerations remain largely unexplored. Although three-dimensional printed models are often described as low-cost solutions, few studies provide detailed analyses of production costs, time requirements, or cost-effectiveness compared with conventional training approaches or digital simulation tools alone.

Future research should therefore move toward controlled, long-term clinical studies able to assess both the safety and the audiological outcomes of these innovations, alongside analyses of their economic sustainability. Standardization of printing protocols, materials, and sterilization procedures will also be crucial for ensuring reproducibility and regulatory compliance. Finally, the expansion of 3D printing toward pharmacological delivery and biofabrication opens the way to a new generation of multifunctional CI, capable of combining electrical stimulation with targeted therapeutic strategies. In this perspective, 3D printing should no longer be viewed merely as a supportive tool, but as a catalyst for a profound transformation of surgical and therapeutic paradigms in otology.

Multicenter studies and shared reporting standards will be essential to determine whether three-dimensional printing can provide measurable clinical benefits in surgical training, preoperative planning, and intraoperative decision-making. Integration with digital planning platforms, robotics, and augmented reality represents a promising direction but will require rigorous clinical validation before routine clinical adoption.

## Conclusions

This systematic review highlights the transformative potential of 3D printing in CI, with applications spanning surgical training, pre-operative planning, and device innovation. The ability to create accurate anatomical models and custom surgical tools has improved precision and educational outcomes while accelerating implant design. Particularly, 3D printing has shown immense value in replicating complex anatomical structures, enabling trainees to familiarize themselves with unique anatomical variations that may arise in real-world cases. This enhances not only technical proficiency but also critical decision-making skills under simulated conditions. As the first systematic review focusing exclusively on CI–specific applications, it addresses a critical gap in the literature. Future research should focus on standardizing methodologies and exploring long-term clinical impacts to further integrate 3D printing into CI practice. Looking ahead, the successful integration of 3D printing into clinical practice for CI will rely on robust multidisciplinary collaborations. The interplay between clinical engineers, otolaryngologists, radiologists, and bioinformaticians will be essential to optimize anatomical models, enhance surgical precision, and accelerate the development of personalized devices.
